# Differential Mutation Detection Capability Through Capture-Based Targeted Sequencing in Plasma Samples in Hepatocellular Carcinoma

**DOI:** 10.3389/fonc.2021.596789

**Published:** 2021-04-30

**Authors:** Jian Gao, Lei Xi, Rentao Yu, Huailong Xu, Min Wu, Hong Huang

**Affiliations:** ^1^ Department of Life Sciences and Technology, Yangtze Normal University, Fuling, China; ^2^ College of Biological Science and Technology, Hubei Minzu University, Enshi, China; ^3^ Department of Infectious Diseases, Southwest Hospital, Third Military Medical University (Army Medical University), Chongqing, China; ^4^ Department of Infectious Diseases, The General Hospital of Western Theater Command, Chengdu, China; ^5^ Chongqing Precision Biotech Co., Ltd., Chongqing, China; ^6^ Institute of Pathology and Southwest Cancer Center, Southwest Hospital, Third Military Medical University, Chongqing (Army Medical University), Chongqing, China

**Keywords:** hepatocellular carcinoma, targeted next-generation sequencing, ctDNA, liquid biopsy, driver genes

## Abstract

Circulating tumor DNA (ctDNA) is a promising biomarker for accurate monitoring and less invasive assessment of tumor burden and treatment response. Here, targeted next-generation sequencing (NGS) with a designed gene panel of 176 cancer-relevant genes was used to assess mutations in 90 ctDNA samples from 90 patients with multiple types of liver disease and 10 healthy donor samples for control. Using our ctDNA detection panel, we identified mutations in 98.89% (89/90) of patient plasma biopsy samples, and 19 coding variants located in 10 cancer-related genes [*ACVR2A, PCLO, TBCK*, adhesion G protein-coupled receptor (*ADGRV1*), *COL1A1, GABBR1, MUC16, MAGEC1, FASLG*, and *JAK1*] were identified in 96.7% of patients (87/90). The 10 top mutated genes were tumor protein p53 (*TP53*), *ACVR2A, ADGRV1, MUC16, TBCK, PCLO, COL11A1*, titin (*TTN*), *DNAH9*, and *GABBR1*. *TTN* and *TP53* and *TTN* and *DNAH9* mutations tended to occur together in hepatocellular carcinoma samples. Most importantly, we found that most of those variants were insertions (frameshift insertions) and deletions (frameshift deletions and in-frame deletions), such as insertion variants in *ACVR2A, PCLO*, and *TBCK*; such mutations were detected in almost 95% of patients. Our study demonstrated that the targeted NGS-based ctDNA mutation profiling was a useful tool for hepatocellular carcinoma (HCC) monitoring and could potentially be used to guide treatment decisions in HCC.

## Introduction

A genetic profile obtained from tumor biopsy specimens is needed for establishing the cancer genome and detecting potentially actionable alterations (1). However, tumor biopsy specimens are invasive and biased and can cause pain and complications for the patient. In addition, it is difficult to perform multiple serial biopsies, and some tumor sites do not lend themselves to biopsy. Moreover, the biopsy specimen might be of too low quality for next-generation sequencing (NGS) in patients who received previous treatment (2–4), and the complete genomic makeup of the malignancy might not be reflected in specimens obtained from the primary site or one metastatic site (5–7).

Mutational analysis is used to determine targeted therapeutic options by identifying resistance mechanisms in patients using repeat tissue biopsies (8). However, it is difficult to obtain adequate tissue specimens for testing in a significant proportion of cases, and therefore the specimen often does not reflect the intra- and intertumoral genetic heterogeneity of the tumor (9, 10). Therefore, a less invasive approach is urgently needed to accurately detect actionable driver mutations and resistance mechanisms.

Several recent studies reported that liquid biopsy could be selected as a routine diagnostic test to provide information on the tumor genome that may allow for personalized medicine (11, 12). Circulating tumor DNA (ctDNA) can be detected in the blood of patients with cancer and sequenced using NGS. CtDNA is a circulating DNA molecule about 150–200 base pairs in size that is released from apoptotic or necrotic tumor cells in primary or metastatic malignant lesions. Therefore, ctDNA reflects the genetic profile of the tumor. Previous studies have demonstrated that ctDNA profiling can be a feasible diagnostic method for disease monitoring, diagnosis, and identification of mechanisms of resistance, such as detecting tumor evolution, response, and resistance (12, 13). CtDNA profiling is especially attractive in patients for whom repeat biopsy cannot be performed because of risk or in whom the quantity/quality of the tissue biopsied is insufficient (13–16). However, previous studies have found that the amount of ctDNA in blood samples is extremely low. Therefore, more sensitive methods are needed to detect mutations in an extremely low amount of ctDNA. Cancer personalized profiling by deep sequencing, which was introduced by Newman et al., used probe hybridization to capture frequently mutated genetic regions, as well as different forms of genetic abnormalities using NGS large-scale parallel sequencing technology. They found that numerous genes contained mutations, fusions, and amplification (15, 16). Identifying different forms of genetic abnormalities through large-scale parallel profiling has proved to be an effective and accurate tool (16).

Hepatocellular carcinoma (HCC) is the third most common cancer and has high cancer-related mortality worldwide. Specific risk factors related to HCC include hepatitis B or C virus infection, high alcohol consumption, and hemochromatosis or non-alcoholic fatty liver disease caused by obesity and insulin resistance (17, 18). But in developing countries like Southeast Asia and sub-Saharan Africa, most HCC cases (60%) occur are hepatitis B virus (HBV)-associated (19). However, the knowledge about genomic alterations implicated in HCC initiation and progression is limited compared with other major lethal types of cancer. Therefore, more efficient methods are needed to develop novel therapeutic agents and strategies for prevention, early diagnosis, and cure. In this study, a ctDNA panel targeting patients with multiple classes of somatic mutations in HCC was designed. The panel contained critical exons and introns located in 176 genes and covered 1,343 single nucleotide variations (SNVs) and 559 insert and deletions (indels) and structural variants (SVs).

## Materials and Methods

### Ethics Statement

All the patients were recruited into the project (NSFC No. 81602606). The studies involving human participants were reviewed and approved by the First Hospital affiliated to the Army Medical University (Southwest Hospital). The patients/participants provided written informed consent to participate in this study. All samples and medical data used in this study have been kept confidential.

### Diagnostic Criteria and Preparation of Plasma Cell-Free DNA

Plasma samples from 90 patients with liver disease were collected and analyzed, of whom 40 were diagnosed with chronic hepatitis B (CHB), 19 with liver cirrhosis (LC), and 31 with primary end-staged HCC from the Department of Infectious Diseases. Alcohol consumption and family history were also assessed. In addition, 10 healthy donor (HD) samples were also collected as controls. CHB cases were defined as hepatitis surface antigen positive, and further classified as active hepatitis B (AHB, liver biopsy shows hepatic activity index by Ishak activity score >3/18 or METAVIR activity score A2 or A3, n = 13) and inactive hepatitis B [IHB, liver biopsy hepatic activity index by Ishak activity score ≤ 3/18 and METAVIR activity score A1, n = 27 (20)]. LC was determined by hepatic ultrasound/abdominal CT/biopsy. HCC was determined by biopsy.

For the preparation of plasma cell-free DNA (cfDNA), peripheral blood (10 ml) was collected at the time of biopsy, stored in tubes with ethylenediaminetetraacetic acid, and incubated for 2 h at room temperature. The supernatant was transferred to centrifuge tubes (15 ml) and centrifuged for 10 min at 16,000 g and 4°C and then stored at 80°C. Circulating cfDNA was collected from plasma (4 to 5 ml) using the QIAamp Circulating Nucleic Acid kit (Qiagen) according to the manufacturer’s instructions. We used the Qubit 2.0 fluorimeter (Thermo Fisher Scientific) for the quantification of cfDNA and Agilent Technologies 2100 Bioanalyzer for analyzing the size distribution of cfDNA. Finally, NGS libraries were constructed using a minimum of 50 ng of cfDNA.

### NGS Library Preparation

Using the KAPA Hyper Prep kit (KAPA Biosystems), sequencing libraries were prepared based on an optimized manufacturer’s protocol. Briefly, 1 μg of genomic DNA was sheared with the Covaris M220 (Covaris), followed by end-repairing, A-tailing, and ligation with indexed adapters, and then size selection was performed using Agencourt AMPure beads (Beckman Coulter, Fullerton, CA, USA). Finally, we used capture probe baits for library hybridization, magnetic beads for hybrid selection, and PCR amplification for identification, as well as purified DNA for target enrichment.

### Panel Selection

The ctDNA panel was based on HBV-associated HCC, as chronic HBV infection remains the major risk factor for HCC in China. The ctDNA panel used sequencing data from The Cancer Genome Atlas (TCGA) and ERP001196, as well as exome sequencing data collected from SRA053063, GSE36390, and GSE62232, and included exons containing recurrent SNVs, indels, and insertions, as well as fusion genes. In this study, regions with a recurrence index of 20 or higher were selected for ctDNA panels, in line with the previous study. In addition, possible targeted therapies for specific characterized genomic alterations were also included, such as beta-catenin (*CTNNB1)*, *MET*, and *EGFR*. Our ctDNA panel covered almost 168 kilobases of 657 human genomic regions spanning 176 cancer-related genes.

### Sequencing Data Analysis

First, Cutadapt (https://pypi.python.org/pypi/cutadapt) and FastQC (www.bioinformatics.babraham.ac.uk/projects/fastqc/) were used to assess the quality of the sequencing data, and then 3′-/5′-adapters and low-quality reads were removed. Using the Burrows–Wheeler Aligner program (bio-bwa.sourceforge.net), the clean reads were collected and mapped to the reference human genome (hg19, genome.ucsc.edu). Alignment files (.bam) were generated by SAMtools (samtools.sourceforge.net), and then low mapping quality reads (<Q30) were filtered out. We also removed clonal duplicated reads using Picard Tools with the default parameters (broadinstitute.github.io/picard/). In addition, using local Perl script, the percentage of alignment of the clean reads to the exome regions was calculated. After that, single nucleotide polymorphisms (SNPs) and indels were called by HaplotypeCaller/Unified Genotyper in GATK (Genome Analysis ToolKit) (www.broadinstitute.org/gatk), using 0.1% as the mutant allele frequency cutoff for liquid biopsy samples. In addition, we used FACTERA (21) and ADTEx (22) to identify gene fusions and copy number variations (CNVs). To identify CNVs, the log2 ratio cutoff for copy number gain was set as 1.6, and a log2 ratio cutoff of 0.67 was used for copy number loss detection in all sample types.

To highlight cancer-relevant alterations and reduce noise from benign germline events, additional custom filtering was applied including 1,000 Genomes (www.1000genomes.org), dbSNP database, ExAC (exac.broadinstitute.org/), ESP6500 (evs.gs.washington.edu/EVS/), and the in-house Chinese Exome Database (1,500 Chinese Han individuals). In brief, we removed frequent germline variants from the 1,000 Genomes Project (snp138NonFlagged), and we highlighted the confirmed somatic alterations that had been deposited in the Catalog of Somatic Mutations in Cancer (COSMIC v70) as biologically significant. The allele frequency cutoffs of short variants present and not-present in COSMIC were set at 1 and 5%, respectively (23). In addition, we removed germline variants with two or more counts (~0.0003% population frequency) in the ExAC database. However, we did keep some known driver germline mutations, such as documented hereditary *BRCA1/2* and tumor protein p53 (*TP53*) deleterious mutations. Moreover, single-nucleotide polymorphisms representing a population frequency higher than 0.1% in the detected variants were excluded from further analysis. We used PolyPhen-2 (genetics.bwh.harvard.edu/pph2/), SIFT (sift.bii.a-star.edu.sg/), and MutationTaster (www.mutationtaster.org/) to identify pathogenic missense mutations.

Here, the number of somatic mutations (both synonymous mutations and non-synonymous mutations) per megabase in each sample was defined as the tumor mutational burden (TMB), excluding hotspot and fusion mutations, indels, splice site mutations and copy number gains and losses (24, 25). We used iCAGEs software to identify cancer driver mutations, genes, and targeted drugs using the somatic mutation profile obtained from our ctDNA panel. Finally, we used ANNOVAR software to annotate the variants, including the consequences, reported allele frequencies, and predicted impacts in the populations (26). Oncoprints were drawn using maftools to visualize the overall mutational landscape (27). A Lollipop plot was constructed for frequently mutated genes to determine the recurrence of genomic loci with variants. Moreover, maftools was used to analyze driver gene detection (oncodrive), mutual exclusive and co-occurring events (somaticInteractions), and pan-cancer comparison (pancanComparison).

## Results

### Patients’ Demographic Characteristics

We collected blood plasma samples from 90 patients and 10 healthy donors. Among them, 63 were men and 27 were women, presented in **Table 1**. Of these, 25 were <40 years old and 65 were ≥40 years old. There were 17 patients with a history of alcohol consumption. There were no significant differences in demographic characteristics among the three groups.

**Table 1:** Clinical Characteristics of circulating tumor DNA detected from enrolled patients.

Characteristics	HCC	LC	CHB	P
n	31	19	40	
Age				0.854
<40	9	6	10	
≥40	22	13	30	
Gender				0.343
Male	24	11	28	
Female	7	8	12	
Alcohol				0.389
yes	4	5	8	
no	5	6	9	
Unknown	22	8	23	

The median cfDNA concentration in HCC patients (164.1 ng/ul) was higher than that in HD group (130.4 ng/ul), but the median cfDNA concentrations in LC (65.15 ng/ul), IHB (76.77 ng/ul), and AHB (69.96 ng/ul) patients were lower than those in healthy donors (**Supplementary Figure 1**). Moreover, the median ctDNA fraction (quantified as the percentage of ctDNA relative to total cfDNA in the sample) was 12.5% (range 0–77.12%) among HDs and higher than other patient groups [HCC: 1.26%, range 0–40%; LC: 2.34%, range 0–75%; IHB: 2.44%, range 0–82%; and AHB: 2.43%, range 0–72%].

### Panel Design and Assessment of NGS Data Quality

In this study, all the 90 plasma samples were used for capture-based targeted deep sequencing using the ctDNA panel to identify somatic mutations. A mean coverage depth of 1,398 was achieved across all target regions of plasma samples, and the percentage of mapped reads was higher than 99.60% among all samples. The mean insert size was 311 bp (**Supplementary Table 1**, **Supplementary Figure 2**).

### Mutation Spectrum in Plasma Samples

**Figure 1:**
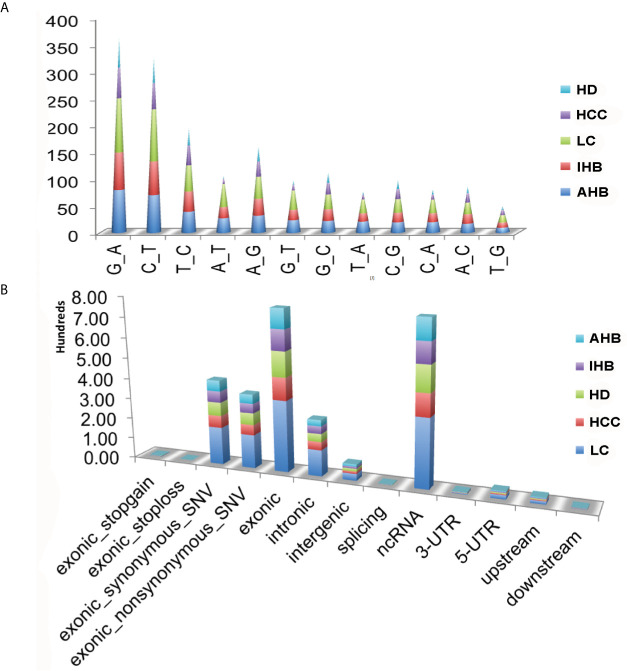
Summary of somatic mutations identified in plasma samples. **(A)** Nucleic acid bases mutations identified in plasma samples, including HCC, LC, IHB, CHB, as well as HD samples. **(B)** Classification of those non-synonymous somatic mutations for plasma samples, including HCC, LC, IHB, AHB, as well as HD samples.

To validate the sensitivity and specificity of the ctDNA panel, plasma samples were used to detect somatic mutations by comparing the results to those of HDs. A total of 1,902 non-synonymous somatic mutations were identified in 167 genes, including 1,343 SNVs, 559 indels, and 33 SVs with CNV signal annotation. Besides, we found that the average frequency of mutations was 14.96 per affected individual, ranging from a minimum of one in an individual with HCC (17R02021) to a maximum of 117 in an LC patient (17R01339) (**Supplementary Table 2**). Notably, among non-silent SNV mutations, the C: G>A: T mutation was significantly enriched in patients with the liver disease compared with HDs. It was most common among patients with LC, followed by AHB, IHB, and HCC patients. The T: A>A: T and G: C>C: G mutations were the second and third most common mutations, respectively (**Figure 1A**).

Classification of the 1,902 non-synonymous somatic mutations showed that most were missense mutations (non-silent) and silent mutations, and most were located in exonic regions or ncRNA (**Supplementary Figure 3** and **Figure 1B**). Patients with LC had the most non-synonymous somatic mutations, followed by patients with AHB, HCC, and IHB. Moreover, the TMB was highest in patients with LC (median: 22) followed by patients with AHB (median: 13), HCC (median: 10), and IHB (median: 6) (**Supplementary Figure 4**).

Additionally, we observed that somatic mutations enriched in 45 cancer­associated genes were shared among HCC, LC, IHB, and AHB patients. Therefore, we compared the mutational spectrum and landscape of those cancer­associated genes with those reported for liver cancer tissues by cBioPortal for Cancer Genomics (including samples from TCGA and ICGC). The 45 queried cancer­associated genes were altered in 1,136 (80%) of the queried patients in the TGCA liver datasets (including PanCan: 377 cases, 89.92%; AMC: 192 cases, 83.1%; Inserm 2015: 192 cases, 79.01%; and MSK 2018: 87 cases, 61.63%) (**Supplementary Figure 5A**). Moreover, progression-free survival and overall survival were calculated in patients with and without alterations in the query genes. The progression-free survival was better in patients without alterations in the query genes (log-rank P < 0.05), but there was no significant difference in overall survival (log-rank P = 0.244). We proposed that the mutational spectrum and landscape of cancer­associated genes identified in our ctDNA panel are important indicators for liver cancer progression (**Supplementary Figures 5B**, **C**).

### Genomic Profiling of Driver Mutations in Patients

**Figure 2:**
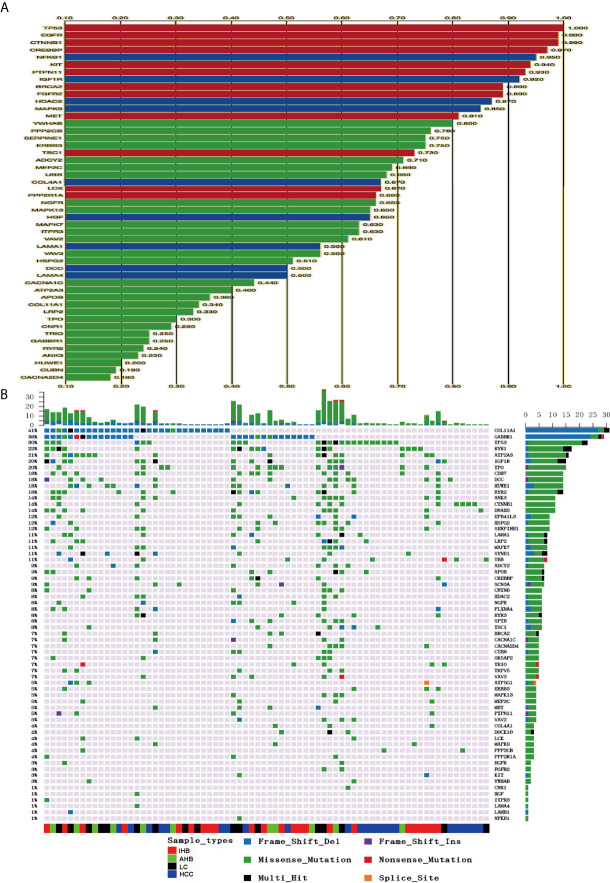
Identification of potential cancer driver genes and their corresponding mutations in plasma samples. **(A)** Potential candidate cancer driver genes were identified using iCAGEs software. **(B)** Summary of mutation detected in candidate cancer driver genes. Different colors of squares represent different types of mutations in the graph. Patient identification numbers are listed at the bottom of the graph. Sample types were divided by different colors of squares on the bottom of the graph. The number of patients having a certain mutation is represented by the bar on the right. The number of mutations that each patient has is represented by the bar on the top of the graph.

Driver mutations tend to be non­silent and are clustered in functionally relevant regions of cancer­associated genes. Potential driver mutations in 62 cancer-associated genes were identified in this study using iCAGEs software (**Figure 2A**). Of those, 12 and nine cancer-associated genes were classified as Cancer Gene Census genes (*TP53, EGFR, CTNNB1, CREBBP, KIT, PTPN11, BRCA2, FGFR2, MET, TSC1, LCK*, and *PPP2R1A*) and KEGG pathway genes *(NFKB1, IGF1R, HDAC2, MAPK9, COL4A1, HGF, LAMA1, DCC*, and *LAMA4*), respectively. In addition, possible therapeutic targets for specific characterized genomic alterations were also identified, such as *EGFR, NFKB1, KIT, HDAC2, MET, TSC1, FGFR2, BRCA2, LCK, SIRT6*, and *ERBB3.* Variants of these 62 potential driver mutation genes were detected in the ctDNA of 76 (84.4%) of 90 patients, including SNVs, indels, fusions, and CNVs **(** **Supplementary Table 3**). Driver mutations in *COL1A1* and *GABBR1* were detected in 41 and 38% of HCC patients, respectively, and were the most commonly mutated genes, followed by *TP53* (30%)*, RYR1* (22%)*, ATP2A3* (21%)*, IGF1R* (20%)*, TPO* (20%*), CDH7* (18%)*, DCC* (18%)*, HUWE1* (18%)*, RYR2* (18%)*, ANK3* (14%)*, CTNNB1* (14%), and *DNAH3* (14%) **(** **Figure 2B** **).** Interestingly, frameshift deletion mutations in *COL1A1* and *GABBR1* were more commonly identified than missense mutations in other driver genes. Frameshift deletion mutations in COL1A1 and GABBR1 may act as important regulators for HCC.

### Genomic Profiling of Frequently Mutated Genes in Patients

**Figure 3:**
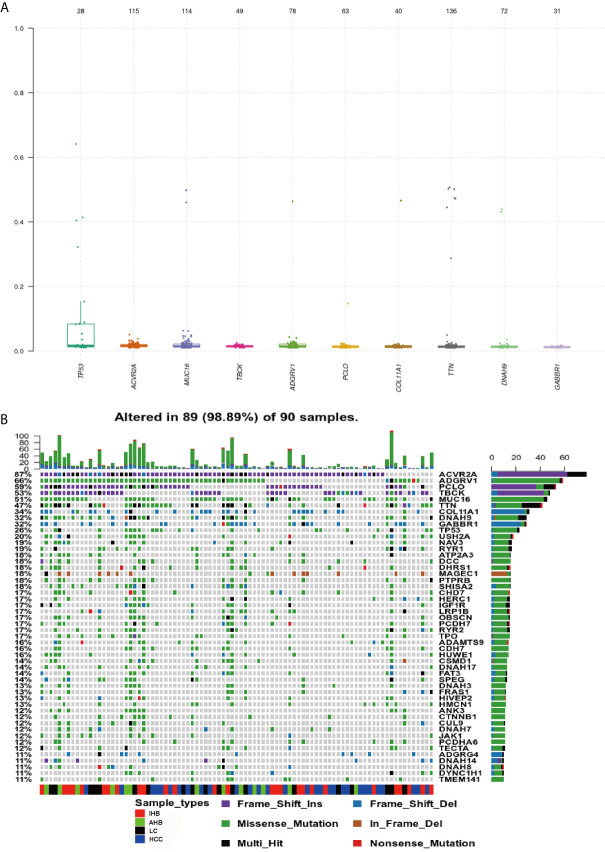
Genomic Profiling of frequently mutated genes and their corresponding mutations identified in plasma samples. **(A)** Top frequent mutated genes identified in plasma samples. A plot of variant allele frequencies as y-axis, top frequent mutated genes as x-axis to estimate the clonal status of top mutated genes. **(B)** Genomic Profiling of mutations in frequently mutated genes identified in plasma samples. Different colors of squares represent different types of mutations in the graph. Patient identification numbers are listed at the bottom of the graph. Sample types were divided by different colors of squares on the bottom of the graph. The number of patients having a certain mutation is represented by the bar on the right. The number of mutations that each patient has is represented by the bar on the top of the graph.

Using our ctDNA panel, mutations were confirmed in 98.89% (89/90) of patient plasma biopsy samples, and the 10 top mutated genes were *TP53, ACVR2A, MUC16, TBCK*, adhesion G protein-coupled receptor *(ADGRV1), PCLO, COL11A1*, titin (*TTN), DNAH9*, and *GABBR1* by assuming pure samples (**Figure 3A**). Of these, we found that mutations in *ACVR2A, PCLO*, and *TBCK* were most frequently frameshift insertions, mutations in *ADGRV1, MUC16, TTN, DNAH9*, and *TP53* were most frequently missense mutations, and *GABBR1* and *COL11A1* mutations were most frequently frameshift deletions (**Figure 3B**).

**Figure 4:**
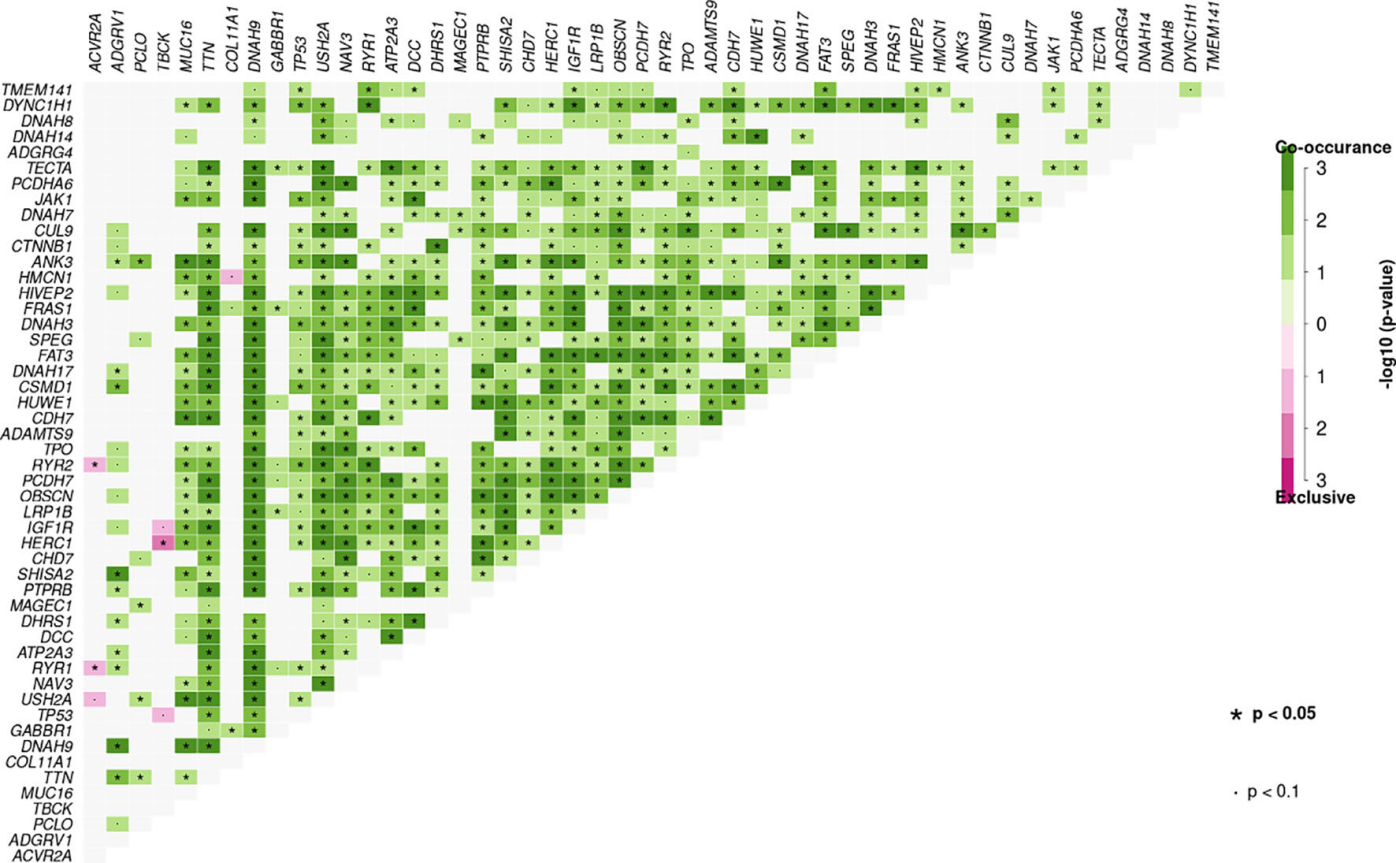
Mutation pattern of co-occurring or show strong exclusiveness between a set of genes in plasma samples. Pair-wise Fisher’s Exact test was used to detect such a significant pair of genes.

Additionally, significant mutually exclusive or co-occurring sets of genes were detected using the somaticInteractions function from maftools and pair-wise Fisher’s exact test. Co-occurring mutations were detected in many sets of genes identified using our ctDNA detection panel. We also observed mutually exclusive mutations in *TBCK* and *HERC* (P < 0.05), *ACVR2A* and *RYR2* (P < 0.05), and *ACVR2A* and *RYR1* (P < 0.05) (**Figure 4**).

Among all genetic aberrations identified, coding variants of *TP53* (p.R43H, p.V25F, p.Y73S, p.M105I, p.R117S, p.P118L, p.I119F, and p.G134R), *TTN* (p.L13269S, p.R14571H, p.Y18131X, p.K23328T, p.L4170S, p.C4883G, and p.T13281fs), *ADGRV1* (p.D1652V, p.T1691M, p.E4479X, and p.E4482V), *CTNNB1* (p.S37A, p.D32G, p.S33P, and p.S33C), transmembrane protein 141 (*TMEM141)* (p.M1L), and ubiquitin B *(UBB)* (p.S65L) occurred in 38, 31, 27, 15, 12, and 12% of 26 individual samples, respectively, of patients with HCC (**Supplementary Figure 6A**). Moreover, somatic mutations in both *TP53* and *TTN* were found in four HCC samples (17R02023, 17R02020, 17R01462, and 17R01446), implying an association between *TTN* and *TP53* mutations (**Figure 4**). Also, the 11 mutations in *TP53* that were present in 10 cases included 11 non-synonymous SNV mutations, and the eight mutations in *TTN* that were present in eight cases included six non-synonymous SNV mutations, one stop-gain, and one frameshift deletion (**Supplementary Figure 6B** and **Supplementary Table 4**).

In 19 individual LC samples, 11, 38, 26, eight, eight, 24, eight, and eight coding variants were detected in *TP53, TTN, ADGRV1, RYR2, RYR1, DNAH9*, *IGF1R*, and *ATP2A3*, respectively, corresponding to 37, 74, 58, 37, 37, 53, 37, and 37% of patients, respectively **(** **Supplementary Figure 7A** **).** Interestingly, many LC patients (42%) carried somatic mutations in both *ADGRV1* and *TTN*, and seven of 19 carried somatic mutations in both *TP53* and *TTN.* Seven of 19 LC patients also carried somatic mutations in both *TP53* and *ADGRV1*, implying associations between *TTN* and *ADGRV1* and between *TP53* and *TTN* mutations (**Figure 4**). In addition, ryanodine receptor family genes, including *RYR1*, *RYR2*, and *RYR3*, were associated in LC patients; 23 coding variants were found in these ryanodine receptor family genes in 15 of 19 LC patients (78.9%) (**Supplementary Table 4**).

Coding variants in *TTN, DNAH9, FRAS1, DCC, MUC16, OBSCN, RYR1, USH2A, ADGRV1, ATP2A3, CHD7, DDX60, DNAH3, DYNC1H1, FAT3, NAV3, PCDH7*, and *SHISA2* were detected in ≥20% of IHB samples **(** **Supplementary Figure 7B**). Moreover, most of these frequently mutated genes were also identified in AHB patients, albeit with different coding variants (**Supplementary Figure 7C**). Coding variants of *DNAH10, DNAH17, DNAH3, DNAH5, DNAH7, DNAH8*, and *DNAH9* were detected in 12 of 27 IHB patients (44.5%) and seven of 13 AHB patients (53.8%) (**Supplementary Table 4**). Besides, many IHB and AHB patients (>30%) carried somatic mutations in both DNAH family genes and *TTN*, implying an association between mutations in *TTN* and DNAH family genes (**Figure 4**).

### Common Mutation Spectra in Plasma Samples

**Figure 5:**
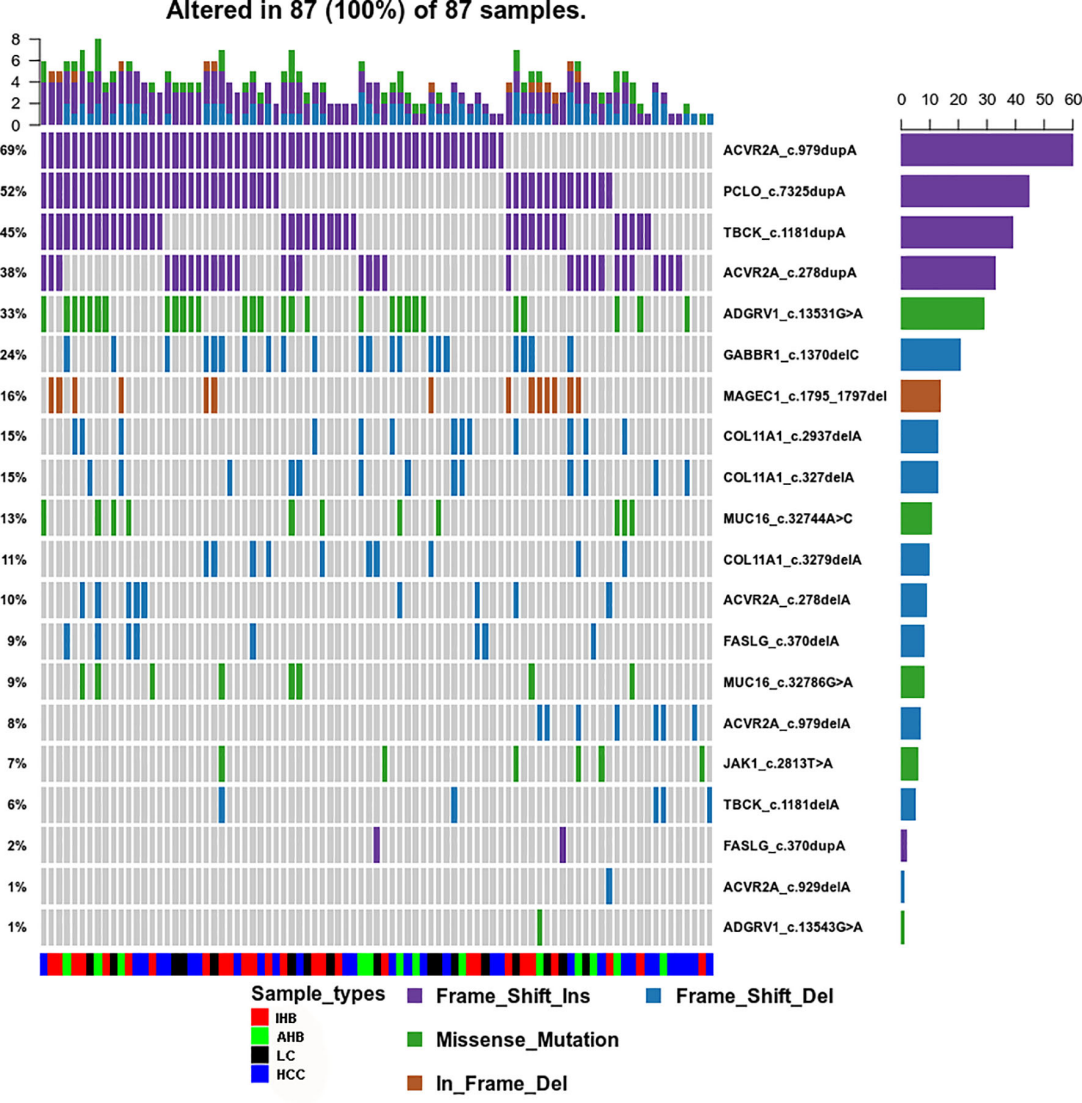
Common Mutation Spectrum of point coding variants identified in AHB, IHB, LC and HCC patients. Different colors of squares represent different types of mutations in the graph. Patient identification numbers are listed at the bottom of the graph. Sample types were divided by different colors of squares on the bottom of the graph. The number of patients having a certain mutation is represented by the bar on the right. The number of mutations that each patient has is represented by the bar on the top of the graph.

To identify the common mutation spectra in plasma samples, venny software was used to access the common coding variants observed in AHB, IHB, LC, and HCC patients. Nineteen coding variants located in 10 cancer-related genes (*ACVR2A, PCLO, TBCK, ADGRV1, COL1A1, GABBR1, MUC16, MAGEC1, FASLG*, and *JAK1*) were identified in 96.7% of patients (87/90). Most importantly, we found that most of those variants included insertions (frameshift insertions) and deletions (frameshift deletions and in-frame deletions). We observed insertion variants in *ACVR2A, PCLO*, and *TBCK* in 95% of patients. Deletion variants in *COL1A1, GABBR1, FASLG*, and *MAGEC1* were also detected. However, missense mutation variants were only found in *ADGRV1, MUC16*, and *JAK1* **(** **Supplementary Table 5** and **Figure 5**).

## Discussion

Identifying genetic changes associated with the development and progression of HCC is vital to improving our understanding of this disease. Although genetic deviations such as genetic mutations in *CTNNB1* and *TP53* have been identified in HCC (28), the overall landscape of genetic alterations during the development and progression of HCC remains unclear. In this study, we performed NGS sequencing using a ctDNA panel and blood plasma samples from patients with AHB, LC, IHB, and HCC to better understand this genetic landscape. We identified mutations in several genes that are often mutated in HCC, including *TP53*, *CTNNB1*, and *TTN*. Also, *TMEM141*, *UBB*, and *ADGRV1* were identified as the most frequently mutated genes in HCC patients.

We identified mutations in *CTNNB1* in 15% of patients with HBV-associated HCC, which is not surprising as *CTTNB1* has been identified as one of the most commonly mutated genes in HCC (29). *TP53* is also frequently mutated or inactivated in HCC (30). We found that 38% of patients with HBV-associated HCC harbored *TP53* mutations. This result is consistent with those of previous studies demonstrating that *TP53* mutations occur in 30–40% of patients with HBV-associated HCC, compared to 20% of patients with HCV-associated HCC (30, 31). We believe that *TP53* mutation might act as a key regulator in HBV-associated HCC. Actually, HCC was molecularly heterogeneous and could be roughly divided into two major subtypes, proliferation subclass and non-proliferation class (32, 33). Specifically, different molecular subclasses or dominant pathways could be identified in different HCC cases, such as telomere maintenance, WNT/*β*-catenin pathway, P53 cell cycle pathway, *etc.* (32). These might explain the reason that different studies reported different mutation abundances. Besides, exome sequencing identifies that different mutations spectra were associated with different HCC risk factors; *CTNNB1* were mainly related to alcohol intake and *TP53* with HBV infection (34). HCC in our study were mainly HBV-related cases, so *TP53* gene was the key mutations in our study.

Distinct mutations in *TP53, TTN*, and *ADGRV1* were identified in LC patients, but only distinct mutations in *TTN* were found in AHB, IHB, and HCC patients. We also found associations between mutations in *TTN* and DNAH family members in AHB and IHB patients, between mutations in *TTN* and RYR family members in LC patients, and between *TTN* and *TP53* mutations in HCC patients. *TTN* encodes a giant protein (>30,000 amino acids) and is rarely recognized as a tumor-associated gene; however, recent studies have suggested that mutations in the highly mutated *TTN* gene are closely related with high TMB status, and the potential biological mechanisms have been elucidated (35). However, our spectral analysis identified distinct mutational spectra at the amino acid level in the development of HCC and significant combinatorial mutational patterns (*TTN* was likely to be co-mutational with other genes during the development of HCC), so its role in the development of HCC still needs to be evaluated.

*UBB* encodes the comprehensive protein degradation signal monoubiquitin and plays a vital role in essential intracellular signaling. The function of ubiquitin is to regulate protein turnover through the ubiquitin/proteasome system. Previous studies showed that the level of ubiquitin reinforces the high metabolic and stress-support system through ubiquitination in cancer cells (36, 37). Moreover, since bortezomib was approved for clinical use by the FDA in 2003, many anticancer drugs have been developed to target various key molecules involved in the ubiquitin–proteasome pathway (38).

However, we have to admit that there were some limitations of our study, like limited samples enrolled, lack of early staged HCC cases and no matched analysis. But the major limitation lies in that no germline DNA data were available. Although a variety of databases and bioinformatics methods were applied, there were still no comparisons with germline samples. In our results, some patients are at a minor allele frequency (MAF) of ~50%, it is possible that these are germline SNPs but not somatic mutations, so we have to admit that some mutations might be a consequence of chromatin immunoprecipitation (ChIP). In addition, ctDNA itself might be useful and have potential for future clinical use, but two major questions should still be studied. One is a better way to extract ctDNA in blood especially in cancers with very low ctDNA content. Another is how to reduce the expensive cost in extraction/storage/sequencing. CtDNA still has a long way to go before it becomes a routine surveillance tool.

In conclusion, we demonstrated mutations in several factors related to HCC that had not been previously identified, such as *TMEM141*, A disintegrin and metalloproteinase with thrombospondin type 1 motif, 9 (*ADAMTS9*), and *ADGRV1*. Of these, *ADGRV1* mutations were most critical, because frameshift deletion variants and frameshift insertion variants were found contained in *ADGRV1*.

## Data Availability Statement

The datasets presented in this study can be found in online repositories. The names of the repository/repositories and accession number(s) can be found below: NCBI BioProject [accession: PRJNA665384].

## Ethics Statement

The studies involving human participants were reviewed and approved by First Hospital affiliated to the Army Medical University. The patients/participants provided their written informed consent to participate in this study.

## Author Contributions

JG and LX designed the research. LX and RY performed the research. HX and JG analyzed the data. MW, RY, and JG conducted experiments. JG wrote the paper. All authors contributed to the article and approved the submitted version.

## Funding

This research is supported by the National Nature Science Foundation of China (NSFC: 81602606).

## Conflict of Interest

HX was employed by the company Chongqing Precision Biotech Co., Ltd. Chongqing.

The remaining authors declare that the research was conducted in the absence of any commercial or financial relationships that could be construed as a potential conflict of interest.
