# Supplementary figures and images for: Differential Mutation Detection Capability Through Capture-Based Targeted Sequencing in Plasma Samples in Hepatocellular Carcinoma
Source: Front Oncol. 2021 Apr 30;11:596789. doi: 10.3389/fonc.2021.596789 (PMC8120297; doi:10.3389/fonc.2021.596789)

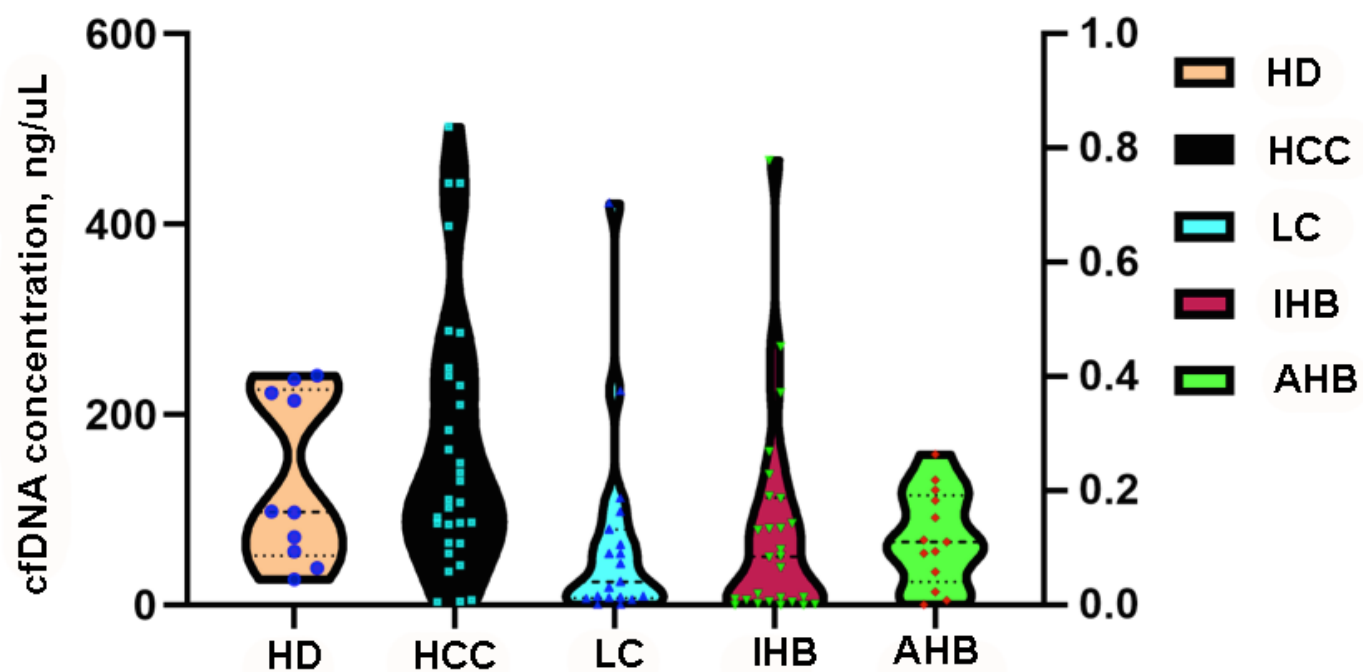

Figure S1. Total of cfDNA concentrations identified in plasma samples.

Supplement: Supplementary file 1 [file DataSheet_1.pdf]
