# Supplementary material for: Differential Mutation Detection Capability Through Capture-Based Targeted Sequencing in Plasma Samples in Hepatocellular Carcinoma
Source: Front Oncol. 2021 Apr 30;11:596789. doi: 10.3389/fonc.2021.596789 (PMC8120297; doi:10.3389/fonc.2021.596789)
Supplement: Supplementary file 2 [file DataSheet_2.pdf]

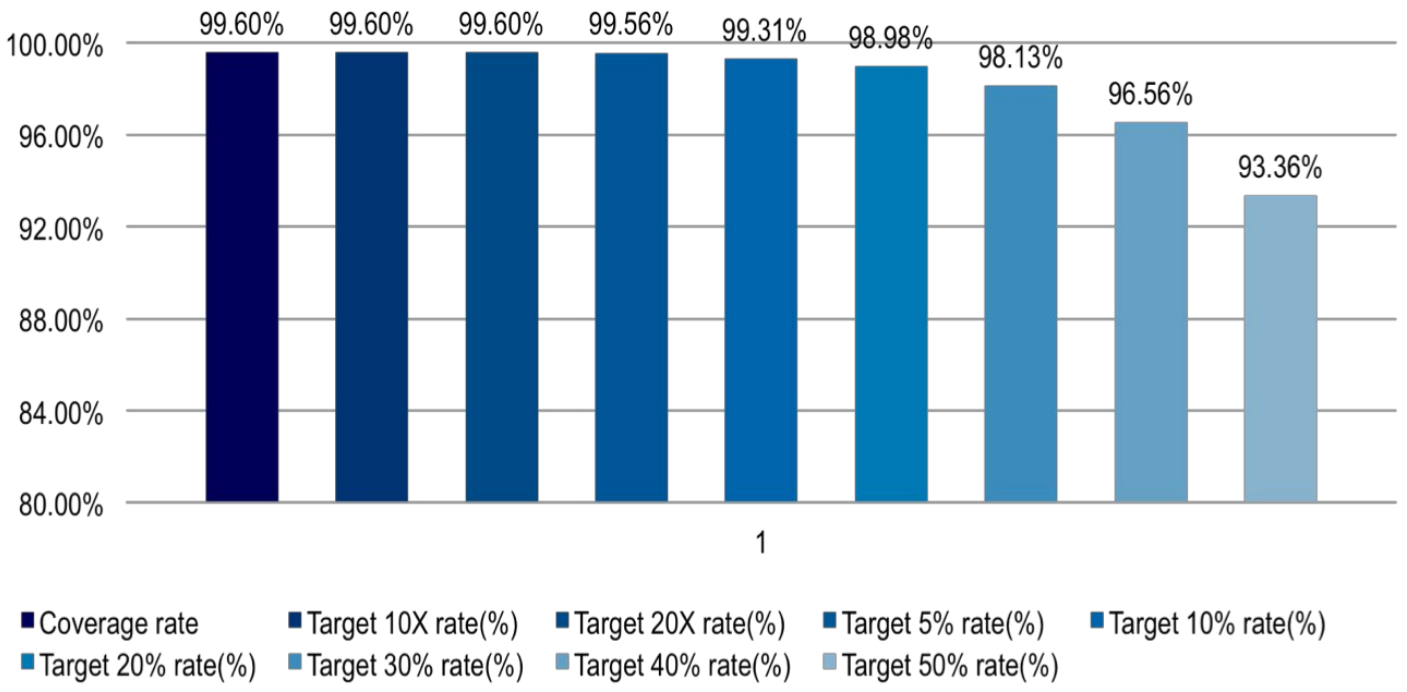

Figure S2. Mean coverage depth, percentage of mapped reads and the mean insert sizes analysis of capture-based targeted deep sequencing using our ctDNA panel for plasma samples.
