# Supplementary material for: Differential Mutation Detection Capability Through Capture-Based Targeted Sequencing in Plasma Samples in Hepatocellular Carcinoma
Source: Front Oncol. 2021 Apr 30;11:596789. doi: 10.3389/fonc.2021.596789 (PMC8120297; doi:10.3389/fonc.2021.596789)
Supplement: Supplementary file 3 [file DataSheet_3.pdf]

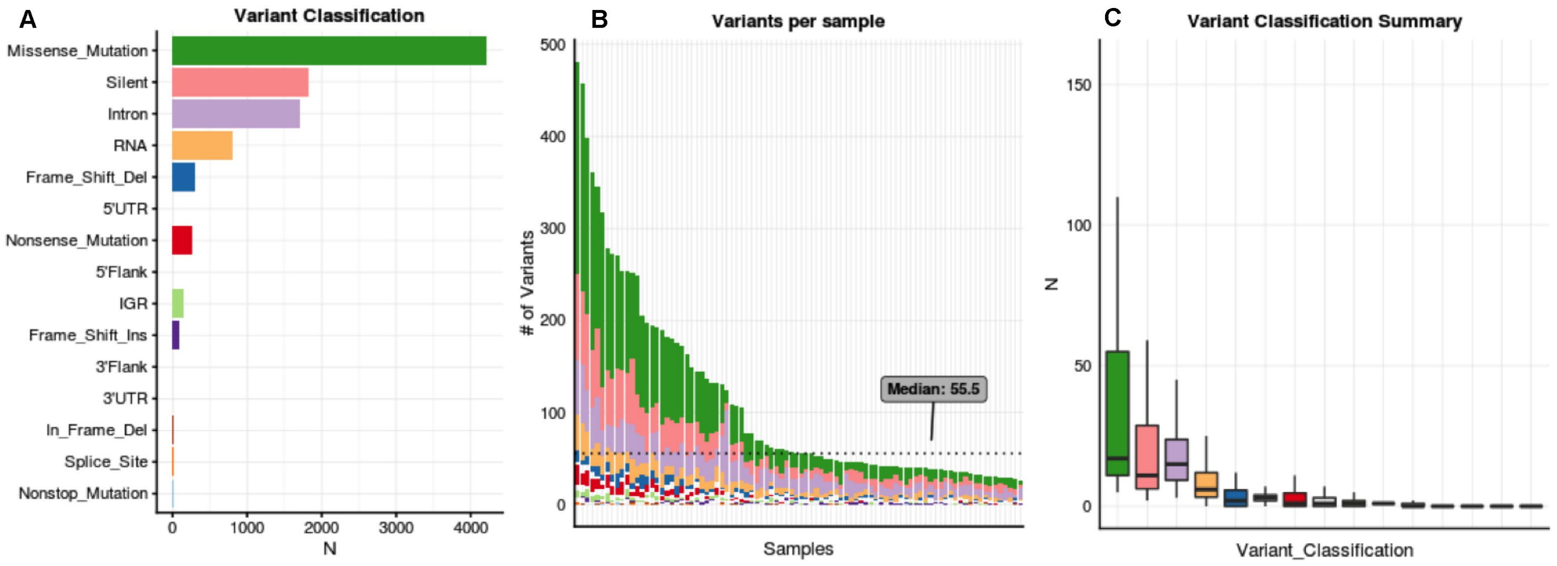


**Figure S3. Summary of mutation information, comprised of variant classification (A), variants per sample (B) and variant classification summary (C) for plasma samples using our ctDNA panel capture-based targeted deep sequencing.**
