# Supplementary material for: Differential Mutation Detection Capability Through Capture-Based Targeted Sequencing in Plasma Samples in Hepatocellular Carcinoma
Source: Front Oncol. 2021 Apr 30;11:596789. doi: 10.3389/fonc.2021.596789 (PMC8120297; doi:10.3389/fonc.2021.596789)
Supplement: Supplementary file 4 [file DataSheet_4.pdf]

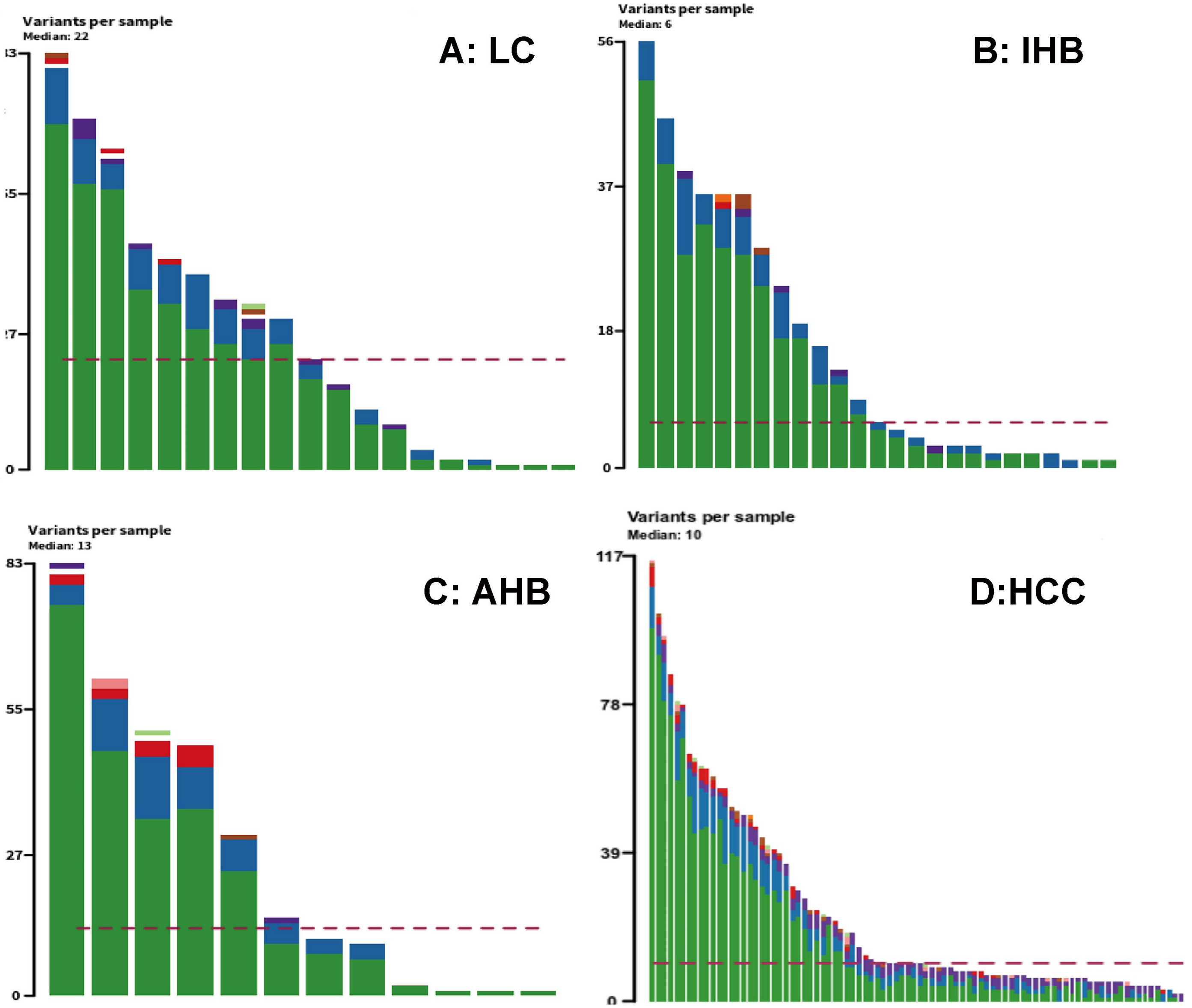

Figure S4. Tumor mutational burden (TMB) for LC (A), IHB (B), AHB (C) and HCC (D) identified from their plasma samples. The number of somatic synonymous mutations per megabase in each sample was defined as TMB.
