# Supplementary material for: Differential Mutation Detection Capability Through Capture-Based Targeted Sequencing in Plasma Samples in Hepatocellular Carcinoma
Source: Front Oncol. 2021 Apr 30;11:596789. doi: 10.3389/fonc.2021.596789 (PMC8120297; doi:10.3389/fonc.2021.596789)
Supplement: Supplementary file 5 [file DataSheet_5.pdf]

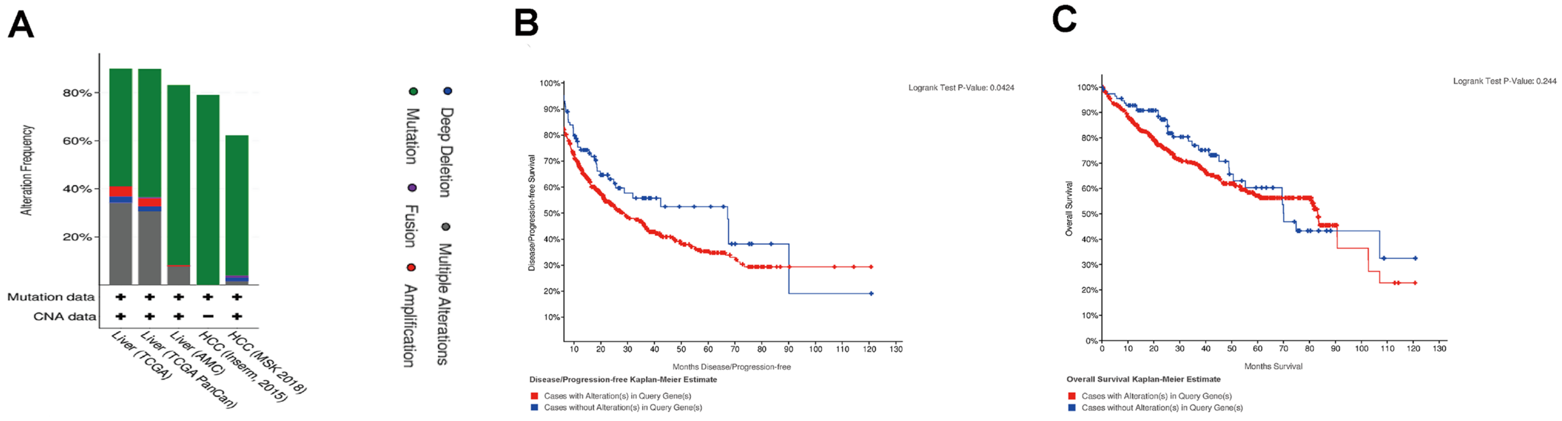

Figure S5. Landscape of candiate cancer-associated genes and survival analysis. (A) Compare the mutational spectrum and landscape of candiate cancer-associated genes with those reported for liver cancer tissues by cBioProtal for cancer Genomics (including samples from TCGA and ICGC). (B) Progression-free survival in the training set calculated with the use of follow-up times (P< 0.05 by the log-rank test) from liver cancer tissues by cBioProtal for cancer Genomics. (C) Overall survival in the training set calculated with the use of follow-up times (P< 0.05 by the log-rank test) from liver cancer tissues by cBioProtal for cancer Genomics.
