# Supplementary material for: Differential Mutation Detection Capability Through Capture-Based Targeted Sequencing in Plasma Samples in Hepatocellular Carcinoma
Source: Front Oncol. 2021 Apr 30;11:596789. doi: 10.3389/fonc.2021.596789 (PMC8120297; doi:10.3389/fonc.2021.596789)
Supplement: Supplementary file 6 [file DataSheet_6.pdf]

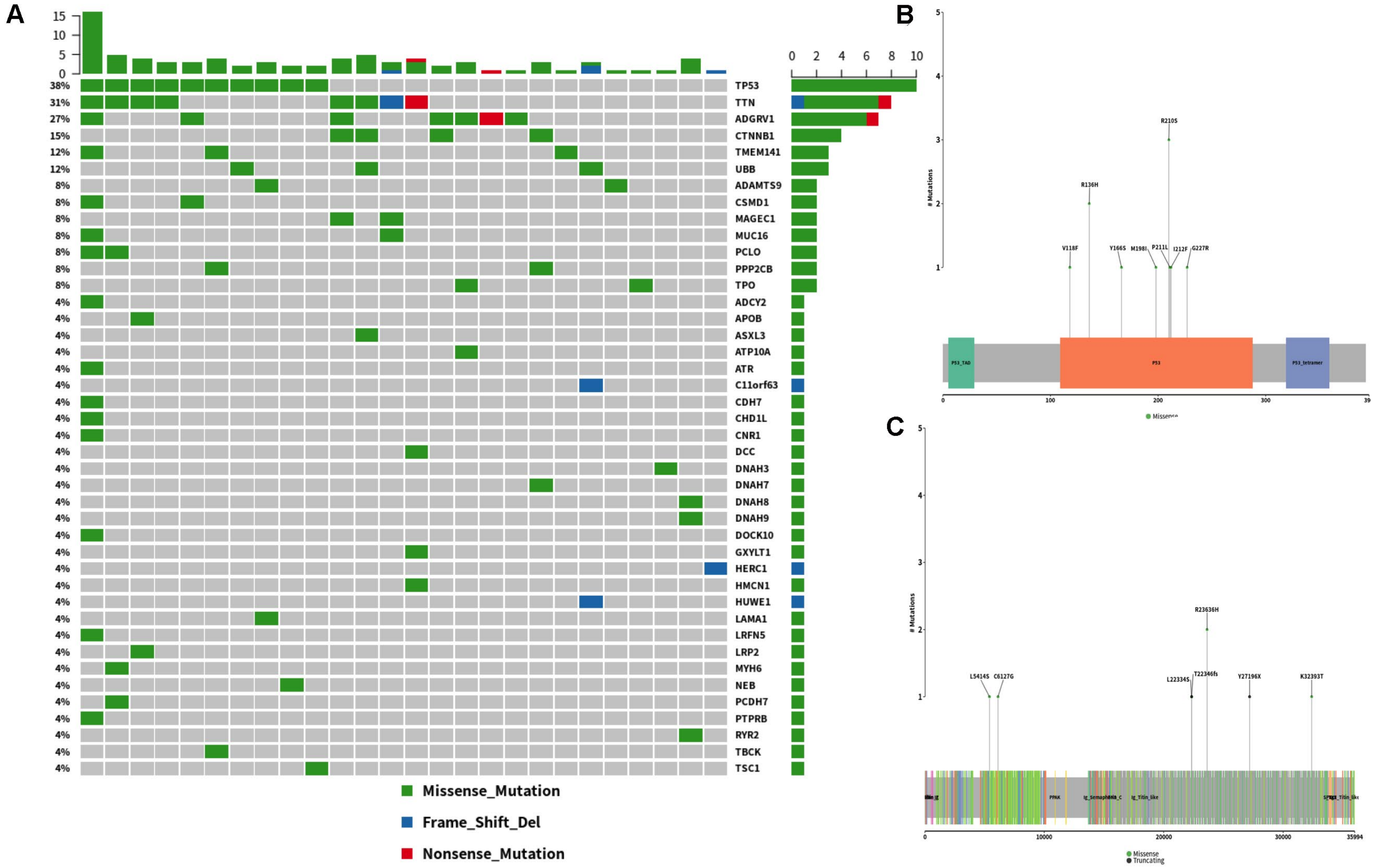

Figure S6. Genetic aberration events and typical mutation details. All genetic aberration events identified for HCC samples, including (A) missense mutation, frame shift deletion, and nonsense mutation. (B) and (C) Summary of mutations located in *TTN* and *TP53* for HCC samples.
