# Supplementary material for: Differential Mutation Detection Capability Through Capture-Based Targeted Sequencing in Plasma Samples in Hepatocellular Carcinoma
Source: Front Oncol. 2021 Apr 30;11:596789. doi: 10.3389/fonc.2021.596789 (PMC8120297; doi:10.3389/fonc.2021.596789)
Supplement: Supplementary file 7 [file DataSheet_7.pdf]

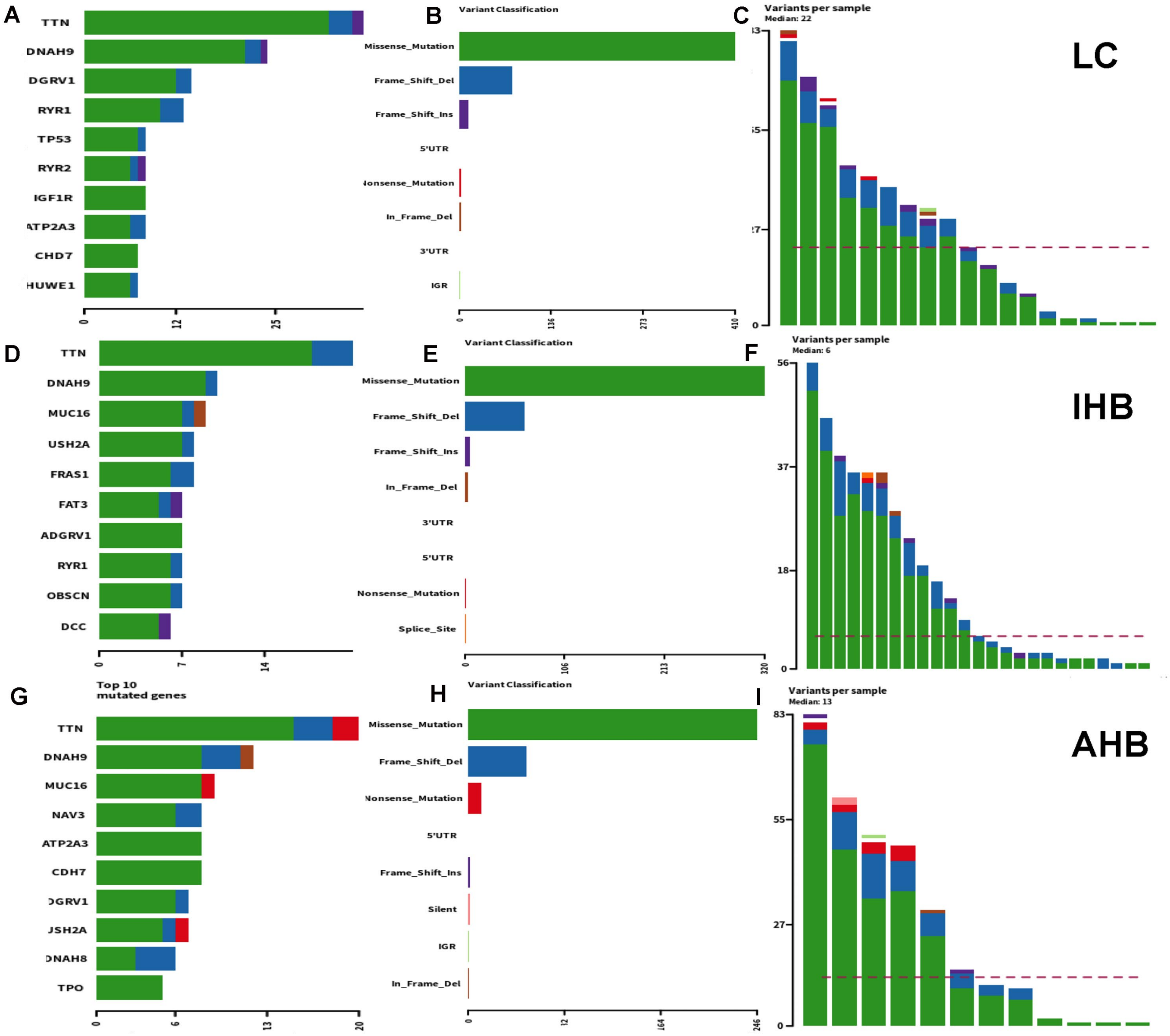

Figure S7. High frequently mutation genes with the different point coding variants identified in patients.

Figures represents high frequently mutation genes with the different point coding variants identified in LC (A/B/C), IHB (D/E/F) and AHB (G/H/I) samples respectively.
